# The interplay of sleep duration, working hours, and obesity in Korean male workers: The 2010–2015 Korea National Health and Nutrition Examination Survey

**DOI:** 10.1371/journal.pone.0247746

**Published:** 2021-03-02

**Authors:** Mi-Jung Eum, Hye-Sun Jung

**Affiliations:** 1 Department of Public Health, Graduate School, The Catholic University of Korea, Secho-gu, Seoul, Republic of Korea; 2 Department of Preventive Medicine, College of Medicine, The Catholic University of Korea, Secho-gu, Seoul, Republic of Korea; University Sains Malaysia, MALAYSIA

## Abstract

The purpose of this study was to clarify the odds ratio for association between working hours and obesity in Korean male wage workers and investigate the role of sleep duration. This study is a cross-sectional one using large-scale national data from the Korea National Health and Nutrition Examination Survey collected between 2010 and 2015 to evaluate 2,592 male wage workers (between the ages of 19 and 60 years). Obesity was defined as 25kg/m^2^ or more and working hours per week were categorized into <40, 40–49, 50–59, and ≥60 hours. Multiple regression analysis was performed to examine the odds ratio for association between working hours and obesity, after controlling for age, education, income, marital status, smoking, drinking, physical activity, daily energy intake, sleep duration, hypertension, diabetes, dyslipidemia, work schedule, and job category. Next, to study the mediating effect of sleep duration on the association between working hours and obesity, an analysis was performed using the Baron and Kenny method and the Sobel test. Results showed that workers with 50 to 59 hours had 1.4 times higher odds (odds ratio [OR] = 1.4, confidence interval [CI]: 1.11–1.85) of obesity and workers with 60 hours or more had 1.4 times higher odds (OR = 1.4, CI: 1.06–1.90) of obesity than workers with less than 40 hours. Sleep was found to have a mediating effect on the association between working time and body mass index. Therefore, the results of this analysis suggest that practitioners should identify potential factors such as working time and sleeping time when preventing work-related obesity.

## Introduction

In 1996, the World Health Organization (WHO) and obesity research reported that obesity increases the risk of diseases such as hypertension, diabetes (especially type 2), coronary heart disease, stroke, and dyslipidemia while also increasing the mortality rate [1–3].

Obesity is defined as an accumulation of fat in the body that is excessive enough to have a negative impact on health [4]. Obesity is a central risk factor in health and has become a public health challenge worldwide [5]. According to the WHO, in 2016, 39% of all adults worldwide were overweight, and 13% of the adult population was obese. This is approximately a three-fold increase between 1975 and 2016 [6]. A previous study in the 2010 National Health Interview Survey of 15,121 employees found that about two-thirds of employed US adults are overweight or obese and 28% are obese [7]. From 1975 to 2014, a study of the trend of changes in BMI in 19.2 million 18-year-olds in 200 countries showed a steady increase in obesity. If this trend continues, it is estimated that the global prevalence of obesity by 2025 will exceed 18% for men and 21% for women [8].

In the Korea National Health and Nutrition Examination Survey (KNHANES), the obesity rate among adults has consistently increased each year [9]. In particular, the prevalence rate of obesity among men has greatly increased over time, and this increase was observed in all age groups [9]. Obesity is socially considered as one of the factors that directly and indirectly increase medical costs and reduce productivity of wage workers [10]. Obese workers were found to have twice the rate of absenteeism, and this tendency was associated with illness [11,12].

Previous studies noted the negative relationship between long duration of work and a range of health problems [9,13–16]. Working long hours is associated with depressive state, anxiety, sleep condition, and coronary heart disease [9], Working more than 52 hours per week had a negative effect on health, regardless of gender [16]. In addition, working beyond the standard hours can increase the risk of obesity, especially in men [17–19]. Since 2018, Koreans have had an average of 2,005 working hours annually, which is 271 hours more than the average annual working hours of 1,734 hours in countries in the Organization for Economic Cooperation and Development (OECD). Further, Korea has held the title of the country with the longest working hours for almost 10 years excluding Mexico [20]. In July 2018, the Korean government implemented a reduction in working hours in phases by amending the labor-related law [21]. According to this law, working hours are defined as fewer than 52 hours per week, including overtime and holiday work; however, this law is currently in a transitional period. These efforts to shorten working hours are expected to promote positive changes regarding various problems caused by longer working hours. Thus, it is necessary to consider the health risks associated with labor time as an important factor at the organizational, social, and national levels.

Sleep has a serious impact on physical and mental health and is another factor affecting obesity. Insufficient sleep is associated with a 1.2 times higher likelihood of obesity and 1.3 times higher likelihood of abdominal obesity [22]. The Odds Ratio (aOR) of obesity was affected more if sleep was insufficient than sufficient [23,24].

Work and sleep are major factors that constitute two-thirds of a day and have a considerable impact on human life. The sleep duration of wage workers can be affected by working hours. In previous studies, cases of working more than 60 hours per week had a 2.1 times higher risk of sleep disorders than those of working less than 40 hours per week [25]. People with long work hours during the workweek had shorter sleep durations during the workweek and the holidays [26]. Wage workers who are obese worked much longer and slept 18 minutes less on average than did wage workers with a normal BMI. Therefore, working hours and short sleep have also been set as the central predictors of obesity [27].

Considering the previously mentioned research studies, we can conclude that an increase in working hours is related to sleep disorders and that working hours and sleep duration have a significant impact on obesity. However, most studies have mentioned independent relationships, and there is insufficient research on the mediating effect of sleep duration on the relationship between working hours and obesity. Therefore, this study aimed to illuminate the relationships between working hours, sleep duration, and obesity in male wage workers, and identify the effect of working hours on obesity while also investigating whether sleep duration plays a mediating role in this relationship.

## Materials and methods

### Study population

We employed a cross-sectional study design using source data of the KNHANES conducted by the National Center for Disease Control in Korea. KNHANES is a survey of health and nutrition at the national level. A 2-level stratified cluster sampling method that used enumeration district and households as the first and second levels of sampling was applied. Health surveys were self-reported. Examinations were conducted through methods such as direct measurement, observation, and sample analysis. The nutrition survey was conducted by a team of specialist surveyors who visited the household and examined it with computer assisted personal interviewing.

The present analysis was conducted using the KNHANES data collected between 2010 and 2015. The initial number of participants was 48,482. We excluded the population without economic activities (*n* = 15,501), women (*n* = 9,263), and those younger than 19 years or older than 60 years (*n* = 2,334). This study was conducted with wage workers who were compensated for their labor by the users; therefore, self-employed, employers, and unpaid family workers were excluded, resulting in a final sample of 5,430 participants.

**Fig 1:**
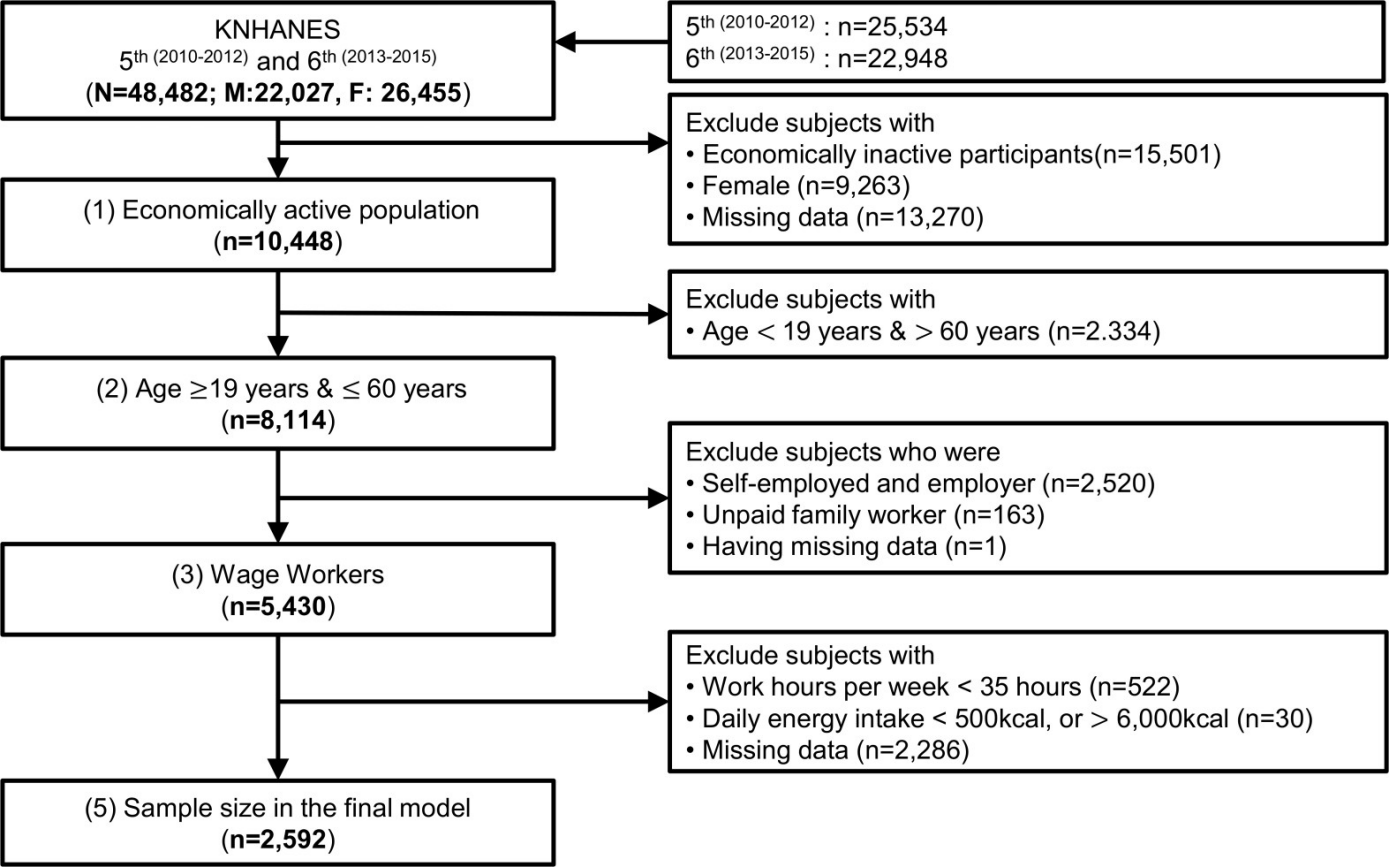
Flow chart of study population.

Furthermore, those working less than 35 hours per week or whose responses on the questionnaire were invalid (daily energy intake >6,000 kcal/day, n = 29; daily energy intake <500kcal/day, n = 1) [28], and having missing data in their survey were not included in this research. Those who work less than 35 hours are generally not likely to work five days a week. As the characteristics of housework have an important effect on the working hours and the sleep duration of women in Korean culture, only male wage workers were included in the analysis in order to intensively explore the effects of working hours and sleep. The final model included 2,592 participants (Fig 1).

### Evaluation of obesity

Typically, obesity is evaluated through BMI, which refers to a value (kg/m^2^) calculated by dividing weight (kg) by the square of height (m). The WHO marks a BMI greater than 25kg/m^2^ as overweight and that greater than 30kg/m^2^ as obesity [2]. However, for obesity standards of Korean adults, the Asia-Pacific WHO Perspective standard of a BMI greater than 25kg/m^2^ is applied [29]. Hence, in this study, obesity is defined as a BMI greater than 25kg/m^2^. Thus, we divided the participants into two groups: the obese group (≥BMI 25kg/m^2^) and the non-obese group (<BMI 25kg/m^2^).

### Questionnaire survey

A questionnaire on general characteristics of participants, such as age (19–29, 30–39, 40–49, 50–60), income (first quartile, second quartile, third quartile, fourth quartile), education level (elementary, middle, high, college or beyond), and marital status (married, divorced or widowed, or single) was administered to the participants. Health-related characteristics of participants included smoking (smoker, ex-smoker, non-smoker), risky alcohol use (average of seven drinks per week for men and five for women, drinking more than twice a week) moderate alcohol use and no alcohol use, physical activity (yes, no), daily energy intake (<2,000, 2,000–2,499, ≥2,500 kcal, which was calculated after conducting a dietary assessment using a 24-hour recall method.), sleep duration per day (<6, 6–7, ≥8 hours), hypertension (yes, no), diabetes (yes, no), and dyslipidemia (yes, no). Occupational characteristics of participants included work schedule such as shift work (day and night shift work, 24-hour shift work), night work (working between 9 pm and 8 am the next day), evening work (working between 2 pm and midnight), and day work (working between 6 am and 6 pm); job category such as professional and office workers (white collar) service and sales workers (pink collar) and functional personnel, machine operator and assembly workers (blue collar); employment status as full-time (employed for more than one year), part-time (temporary employment), and day-to-day (employed on a daily basis); and working hours per week (<40, 40–49, 50–59, ≥60 hours).

### Statistical analysis

All data were processed by designing a complex sample where weighted frequencies were calculated by computing weight guidelines recommended by the Korea Centers for Disease Control and Prevention. Descriptive statistics were used to summarize the general, health-related, and occupational characteristics of the participants. Categorical variables are expressed as frequencies and percentages, and continuous variables as mean and standard deviation. To analyze factors related to obesity, categorical variables were tested using χ^2^, and continuous variables were tested using the *t*-test.

The relationship between occupational characteristics and sleep duration, based on working hours, within the groups was additionally examined using χ2. Using multiple logistic regression analysis, the odds ratio for association between working hours and obesity was examined after controlling for individual variables.

Model I adjusted for the effects of age, education level, income, and marital status. Model II additionally adjusted for the effects of smoking, alcohol use, physical activity, daily energy intake, employment status, job category, work schedule, hypertension, diabetes, and dyslipidemia. A final analysis was conducted with Model III by further adjusting for sleep duration on Model II. The adjusted aOR and 95% confidence interval (CI) were calculated.

There are two ways to verify the mediating effect: the Baron & Kenny method [30] and the Sobel test [31]. Analyzing the hierarchical change of Baron and Kenny is performed in three stages, and the effects of independent variables on mediating variables are verified in Step 1. In Step 2, the effects of independent variables on dependent variables are analyzed, and in Step 3, the effects of independent variables and mediating variables are analyzed simultaneously on dependent variables. As a result, if the effects of independent variables on parameters and dependent variables are significant in Steps 1 and 2, and if the effects of independent variables decrease by more than two levels in Step 3, the mediated effects of mediating variables can be determined to be significant. Therefore, to study the mediating effect of sleep duration on the association between working hours and obesity, a hierarchical regression analysis was performed using the Baron and Kenny method. Additionally, the Sobel test was conducted by applying a Sobel formula,

$$(\mathrm{Sobel}\;Z=(\mathrm{AB})/\sqrt{{\left(A*SE_{B}\right)}^{2}+{\left(B*SE_{A}\right)}^{2})}$$

to determine the significance of the mediating effect by deriving a significant probability (*p*-value) for the mediated effect based on regression coefficient and standard error. All statistical analyses were performed on SPSS (version 23, IBM Corp., Armonk, NY, USA), and two-tailed *p*-values less than .05 were considered statistically significant.

### Ethical considerations

This survey was conducted by the Korea Center for Disease Control. The participants gave signed consent. Participants were treated anonymously before data analysis was conducted. This study was conducted after receiving approval from the Institutional Review Board of The Catholic University of Korea (MIRB-MYUN20190524-002).

## Results

Table 1 shows the relationship between general, health-related, and occupational characteristics between the obese and non-obese groups using χ2. The mean age±standard error (SE) of the participants was 39.4±0.168. The mean working hours per week±SE was 49.3±0.122 hours, and the mean sleep duration per day±SE was 6.7±0.011 hours. A total of 1,012 participants (39.0%) were considered obese. Men who smoked, were high-risk alcohol drinkers, or lacked adequate sleep had higher rates of obesity. Men with high daily energy intake or those with hypertension, diabetes, and dyslipidemia had higher rates of obesity. Obesity was more common among those who engaged in shift work and white-collar workers were more obese. As working hours increased, the obesity rate rose. The chi-square test showed that the distribution of variables such as age, education level, income, smoking, drinking, physical activity, sleeping time, daily energy intake, hypertension, diabetes, dyslipidemia, work schedule, employment status, and job category, differed significantly between the obese and non-obese groups.

**Table 1:** Prevalence of general and occupational characteristics in obese and non-obese participants.

Variables	(n = 2,592)		
	Obese (n = 1,012)	Non-obese (n = 1,580)	*p*-value
Age (yrs)*		39.4±0.168	
19–29	102 (32.1)	212 (67.9)	< .001
30–39	358 (41.1)	521 (58.9)	
40–49	341 (44.5)	443 (55.5)	
50–60	211 (33.7)	404 (66.3)	
Education			
Elementary	19 (26.1)	56 (73.9)	.001
Middle Low	57 (40.9)	89 (59.1)	
Middle High	351 (39.5)	539 (60.5)	
High	585 (39.4)	896 (60.6)	
Income			
First Quartile	27 (25.0)	62 (75.0)	.001
Second Quartile	226 (38.0)	362 (62.0)	
Third Quartile	383 (41.1)	575 (58.9)	
Fourth Quartile	376 (39.4)	581 (60.6)	
Marital status			
Married	820 (41.1)	1,215 (58.9)	< .001
Divorce or widowed	29 (41.2)	45 (58.8)	
Single	163 (32.3)	320 (67.7)	
Smoking			
Smoker	440 (40.4)	658 (59.6)	.004
Ex-Smoker	327 (40.4)	499 (59.6)	
Non-Smoker	245 (35.7)	423 (64.3)	
Alcohol Use			
Risky	260 (47.1)	302 (52.9)	< .001
Moderate	558 (38.3)	915 (61.7)	
No	194 (33.5)	363 (66.5)	
Physical activity			
Yes	547 (40.5)	793 (59.5)	.025
No	465 (37.6)	787 (62.4)	
Sleep duration(h)*		6.7±0.011	
<6	111 (42.3)	154 (57.7)	.038
6–7	685 (39.1)	1,078 (60.9)	
≥8	216 (37.7)	348 (62.3)	
Daily Energy Intake (kcal)*		2,555.1±12.473	
<2000	302 (40.6)	452 (59.4)	< .001
2000–2499	216 (30.5)	444 (69.5)	
≥2500	494 (42.7)	684 (57.1)	
Hypertension			
Hypertension	364 (60.6)	256 (39.4)	< .001
Pre-Hypertension Stage	369 (44.4)	491 (55.6)	
Normal Blood Pressure	289 (24.6)	833 (75.4)	
Diabetes			
Diabetes	91 (52.6)	88 (47.4)	< .001
Impaired Fasting Glucose	313 (54.3)	275 (45.7)	
No Diabetes	608 (33.5)	1,217 (66.5)	
Dyslipidemia			
Yes	574 (54.0)	520 (46.0)	< .001
No	438 (29.0)	1,060 (71.0)	
Work schedule			
Shift	129 (41.2)	176 (58.8)	< .001
Night	20 (39.2)	28 (60.8)	
Evening	23 (25.1)	56 (74.9)	
Day	840 (39.5)	1,320 (60.5)	
Employment status			
Full-time	905 (39.8)	1,384 (60.2)	.001
Temporary	64 (32.4)	119 (67.6)	
Day-to-day	43 (40.7)	77 (59.3)	
Job Category*			
Blue Collar	349 (37.2)	587 (62.8)	< .001
Pink Collar	99 (34.2)	177 (65.8)	
White Collar	564 (41.6)	816 (58.4)	
Working hours/week*		49.3±0.122	
<40	54 (33.6)	107 (66.4)	.037
40–49	526 (39.0)	833 (61.0)	
50–59	255 (40.7)	374 (59.3)	
≥60	177 (39.6)	266 (60.4)	

*Values are Mean ± SE (standard error); BMI: Body mass index; Non-obese: BMI (kg/m2) <25, Obese: BMI (kg/m2) ≥25). Job category was defined as *white collar*, professional and office workers; *pink collar*, service and sales workers; *blue collar*, functional personnel, machine operators, and assembly workers).

Table 2 shows occupational characteristics and sleep duration by groups based on working hours. According to the chi-square test, there was a significant difference in occupational characteristics and sleep duration in the working hour groups. Workers with more than 60 working hours were shown to have insufficient sleep.

**Table 2:** Occupational characteristics and sleep duration by the groups based on working hours.

Variables	Working hours/week				*p*-value
	<40 (n = 161)	40–49 (n = 1,359)	50–59 (n = 629)	≥60 (n = 443)	
Work schedule					
Shift	17 (5.0)	126 (41.8)	67 (23.2)	95 (30.1)	< .001
Night	5 (10.5)	14 (27.2)	12 (24.2)	17 (38.1)	
Evening	17 (28.3)	31 (35.3)	14 (16.6)	17 (19.8)	
Day	122 (5.9)	1,188 (54.1)	536 (24.5)	314 (15.5)	
Employment status					
Full-time	123 (5.6)	1,218 (52.1)	562 (24.3)	386 (18.0)	< .001
Temporary	23 (14.5)	78 (43.0)	43 (24.9)	39 (17.6)	
Day-to-day	15 (11.0)	63 (56.1)	24 (18.5)	18 (14.4)	
Job category					
Blue Collar	51 (5.5)	421 (44.3)	248 (26,6)	216 (23.8)	< .001
Pink Collar	31 (13.4)	125 (42.2)	65 (23.6)	55 (20.8)	
White Collar	79 (6.1)	813 (59.0)	316 (22.3)	172 (12.7)	
Sleep duration(h)					
<6	12 (5.0)	117 (42.1)	69 (25.6)	67 (27.3)	< .001
6–7	105 (6.4)	943 (53.3)	426 (24.1)	289 (16.2)	
≥8	44 (8.4)	299 (50.6)	134 (23.1)	87 (18.0)	

Table 3 shows the results of the logistic regression analysis on the odds ratio for association between working hours and obesity.

**Table 3:** Association between work hours and obesity of study participants.

Working hours per week	Model I		Model II		Model III	
	OR (95%CI)	*p*-value	OR (95%CI)	*p*-value	OR (95%CI)	*p*-value
<40	Reference		Reference		Reference	
40–49	1.2 (1.01–1.42)	.037	1.2 (0.97–1.51)	.094	1.2 (0.97–1.50)	.096
50–59	1.3 (1.06–1.62)	.014	1.4 (1.12–1.86)	.005	1.4 (1.11–1.85)	.007
≥60	1.3 (0.98–1.62)	.069	1.4 (1.07–1.92)	.018	1.4 (1.06–1.90)	.020

*Adjusted ORs from multivariate logistic regression analysis. Model I: Adjusted for age, education, income, marital status; Model II: Adjusted for Model I + smoking, alcohol use, physical activity, daily energy intake, hypertension, diabetes, dyslipidemia, work schedule, employment status, job category; Model III: Adjusted for Model II + sleep duration.

The results of the final analysis showed that in Model III, while the odds of obesity in those working 40–49 hours per week was not significantly higher than in those working less than 40 hours, the odds of obesity in those working 50–59 hours per week was 1.4 times higher (OR = 1.4, CI: 1.11–1.85, *p* = 0.007) and the odds of obesity in those working 60 hours or more was about 1.4 times higher (OR = 1.4, CI: 1.06–1.90, *p* = 0.020).

Table 4 shows sleep duration mediating role on the effect of working hours on BMI. In the Baron and Kenny hierarchical regression analysis, Model I verified the effect of working time on sleep time and Model II verified the effect of working time on obesity. Finally, the effect of working time and sleep time on BMI was analyzed in Model III. We used the results to judge whether working time had a mediating effect of sleep duration.

**Table 4:** Mediating effect of sleep time using Baron and Kenny method & Sobel test.

	Step Ⅰ		Step Ⅱ		Step Ⅲ
	sleep duration		BMI		BMI
**Constant**	6.9***		21.4***		22.0***
Working Hours	-0.012***		0.006*		0.005
Sleep Duration					-0.096**
***R*^2^**	.042		.222		.223
***F***	155.9***		262.0***		534.6***

*p< .05

** *p* < .01

*** *p* < .001.

Sobel *Z* = 2.7, *p* = .006

In Step 1, there was a significant negative effect of working hours on sleep duration (*B*±SE = -0.012±0.001, *p*<0.001). In Step 2, there was a significant positive effect of working hours on BMI (*B* = 0.006±0.002, *p* = 0.01), with BMI increasing as working hours increased. In Step 3, after adjusting for the effect of sleep duration, working hours did not continue to have a significant association with BMI, but it was found that sleep duration was negatively associated with BMI (*B* = -0.096±0.035, *p* = 0.08). According to the analysis, sleep duration was found to have full mediation.

Additionally, the Sobel test was analyzed based on the relationship between working hours and sleep duration and the relationship between sleep duration and obesity. The results of the Sobel test showed Sobel’s *Z* value was significant (*Z* = 2.7, *p* = 0.006). In conclusion, this finding provides the evidence that sleep duration has a mediating effect on the relationship between working hours and BMI.

## Discussion

In this study, sleep duration was found to intensively mediate the relationship between working hours and BMI. Partly explaining the interplay between obesity, sleep and working hours, there was a significant negative effect of working hours on sleep duration, and there was a significant positive effect of working hours on BMI with BMI increasing as working hours increased. However, in the final model, working hours did not continue to have a significant association with BMI, but it was found that sleep duration was negatively associated with BMI. In other words, longer working hours appear to contribute to insufficient sleep, which leads to the development of obesity.

Various studies investigated the relationship between working hours and insufficient sleep [32,33] and found that longer working hours were related to shorter sleep duration and reduced quality of sleep. In a study on middle-aged Australian adults, Magee, Caputi, and Iverson [34] reported that insufficient sleep partially mediated the relationship between working hours and BMI in male participants [34]. This previous study’s [34] generalizability is limited as it had only middle-aged adults as participants and the analyses were conducted by excluding job-related factors, such as work schedule and job category. In contrast, the present study can be significant because it was conducted with wage workers, irrespective of age, and the results were calculated after adjusting for obesity-related variables, such as job-related factors.

In the current study, the working hours of male wage workers were related to the risk of obesity. The relationship of obesity with demographic, health-related, and occupational factors was evaluated, and significant relationships were found with all variables. Furthermore, the results after adjusting for these confounding variables using multiple regression showed that the OR of obesity in men who worked for 50–59 hours per week was 1.4 (95% CI, 1.11–1.85, *p* = 0.007), and the OR of obesity in men who worked for ≥60 hours per week was 1.4 (95% CI, 1.06–1.90 *p* = 0.020), compared with those working for <40 hours per week.

These results are similar to the results of a previous study on 15,121 American adult wage workers that found that the risk of obesity was 1.3 higher for those working more than 50 hours than those working less than 30 hours per week [7]. There can be various explanatory reasons for the presence of a relationship between working hours and obesity. Long working hours can hinder various activities, such as exercise, sleep, leisure activity with family, and meeting social needs, and can function as a factor that induces metabolic problems, such as obesity [17,35]. Insufficient sleep is related to inducing changes in the leptin and ghrelin hormones and can induce changes in the metabolism, thereby contributing to sleep disorders as well as an increase in the BMI [36]. Taheri et al. [36] have reported that the mean change rates of weight increase and sustained sleep duration have a negative correlation. Although wage workers with long working hours require more time for recovering from fatigue due to work [37], long working hours can be said to hinder this.

Weight can be affected by the interaction between genetic, environmental, and psychosocial factors that function through the physiological control of energy intake and consumption and physical activity [38]. Lifestyle can contribute to the prevalence of obesity. Although employers have pursued various strategies as an effort to manage the health of the wage workers, it is important to focus on health management that aims to reduce risks related to major lifestyle patterns of wage workers [39].

The obesity of wage workers can be seriously considered along with revisable risk factors, such as unhealthy habits and drug treatments [3]. Considering that an effective approach to address the problem of wage workers’ weight might be limited, further effort is needed to identify and control revisable latent factors, such as working hours and sleep duration, to lead to methods of preventing wage workers’ obesity [40]. Therefore, effective interventions to prevent obesity are necessary not only on the individual level, but also the organizational level. Observing working hours and rest periods in the workplace and healthy eating, physical activity, and improvement of quality of sleep should serve as a means of improving workers’ health.

The major strength of this study was that it showed the relationship between working hours and obesity and identified the mediating effect of sleep duration on the relationship between working hours and obesity by using a national large-scale sample. One of the main limitations is that these results cannot necessarily be generalized to other populations or the general public, given that it was studied specifically for the selected population of workers in Korea. In addition, while studies of female obesity as well as men are valuable, we only studied men. Women have hormone-related factors such as pregnancy, childbirth, and menopause that play a role in obesity [41]. Women are also more likely to work and be the primary caregiver for children than men, which can affect their sleep. This trend is especially strong for women in Korea. Moreover, given the nature of self-report, working hours and sleep duration may have been inaccurate.

Furthermore, cross-sectional surveys cannot identify the causal relationship between working hours and obesity; therefore, further studies using a longitudinal methodology are required to understand the causal relationship between these variables.

## Conclusions

This study showed that working hours and obesity are related after adjusting for confounding variables, such as occupational and health-related characteristics, which can affect obesity and that insufficient sleep can have a mediating effect on the relationship between working hours and obesity. Therefore, these results suggest that it is useful to identify potential factors such as working and sleeping hours in preventing and managing obesity in wage workers.
